# Anginal Symptoms in Patients With Type 2 Myocardial Infarction: Insights From the TARGET-Type 2 Trial

**DOI:** 10.1016/j.jacadv.2026.102630

**Published:** 2026-02-20

**Authors:** Simran Piya, Caelan Taggart, Amy Ferry, Jagdeep S. Singh, Anny Briola, Nicholas L. Mills, Andrew R. Chapman

**Affiliations:** a BHF Centre of Research Excellence, University of Edinburgh, Edinburgh, United Kingdom; b Victoria Hospital, Kirkcaldy, United Kingdom; c Edinburgh Clinical Trials Unit, University of Edinburgh, Edinburgh, United Kingdom; d Usher Institute, University of Edinburgh, Edinburgh, United Kingdom

**Keywords:** coronary artery disease, type 2 myocardial infarction, universal definition

**What is the clinical question being addressed?**

What symptoms do patients with type 2 MI experience and does this inform targets for future treatment?

**What is the main finding?**

Almost half of all patients with type 2 MI experience angina in the 3 months prior to their acute event, which may represent an important target for treatment.

The Fourth Universal Definition states a myocardial infarction (MI) occurs where there is evidence of acute myocardial injury (defined by a rise and or fall in cardiac troponin concentration), alongside symptoms or signs of myocardial ischemia or imaging evidence of loss of viability.^1^ A type 1 MI occurs due to atherosclerotic plaque rupture or erosion with thrombosis, whereas a type 2 MI occurs without thrombosis, due to an imbalance in myocardial oxygen supply or demand due to another acute illness, or an alternative acute coronary pathology. Patients with type 2 MI have poor outcomes, with as few as one in 3 alive at 5 years, and over two-thirds of patients have unrecognized coronary artery disease, which has been shown to be associated with future adverse cardiovascular outcomes.^2^ However, due to the heterogeneity inherent in this condition, no definitive randomized controlled trials have been conducted, and there is limited evidence to guide clinicians in practice. Furthermore, there are no data on patient symptoms before or after type 2 MI, which may be informative in defining patient-centered targets for treatment. The aim of the current study is to define anginal symptoms in patients with type 2 MI who were enrolled in the TARGET-Type 2 MI (Targeting Investigation and Treatment in Type 2 Myocardial Infarction; NCT05419583) study.

## Methods

TARGET-Type 2 MI was a pilot, prospective, randomized controlled trial which focused entirely on patients with type 2 MI and aimed to implement a complex intervention of targeted investigation and treatment for coronary or structural heart disease.^3^ This trial aimed to demonstrate the feasibility of screening, recruitment, and randomization of patients with type 2 MI to inform a definitive clinical trial. The primary outcome was the proportion of patients identified as eligible who were screened, recruited, randomized, and completed follow-up.

Patient interviews were conducted at the time of enrollment to document symptom burden prior to their type 2 MI event, in order to define the relevance of anginal symptoms as a patient-reported outcome in future clinical studies. Anginal symptoms were assessed using the shortened rose angina questionnaire and recorded prospectively in a REDCap database within a secure data environment. This study was approved by the North of Scotland Research Ethics Committee (REC: 22/NS/0085) and carried out in conjunction with the Edinburgh Clinical Trials Unit. All data analysis was conducted using R (version 4.4.1).

**Figure 1:**
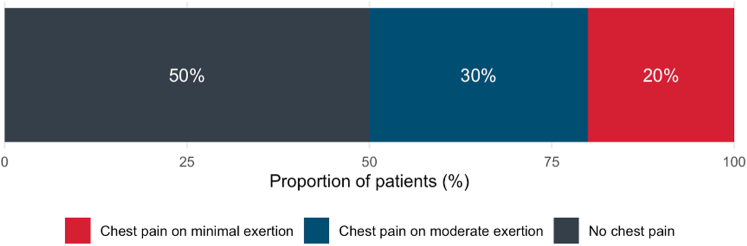
**Anginal Symptoms After Type 2 MI** Summary of anginal symptoms in the 3 months prior to type 2 MI in TARGET-type 2 MI trial participants (n = 60). MI = myocardial infarction.

During a 12-month screening period, 143 patients (35%) were eligible, 119 patients (83%) were approached, and 60 patients (42%, age 70 ± 10 years, 38% [23/60] women, 25% [15/60] known coronary artery disease) randomized to the intervention (n = 28) or standard care (n = 32). The most common causes of type 2 MI were tachyarrhythmia (47% [28/60]), hypoxemia (22% [13/60]), and anemia (8% [5/60]). We found 48% (29/60) of patients experienced chest pain or discomfort in the 3 months prior to type 2 MI. In this group, 20% (12/60) experienced chest pain on minimal exertion when walking at an ordinary pace on the level, and 30% (18/60) experienced chest pain on moderate exertion when walking uphill or hurrying (Figure 1). We found 12% (7/60) of patients were unable to walk or hurry due to another functional limitation. Most patients presented with chest discomfort (33%, 20/60), breathlessness (23%, 14/60), or palpitation (17%, 10/60), and 30% (18/60) of patients experienced 2 or more episodes of chest pain in the 24 hours prior to admission.

## Discussion

Coronary artery disease is increasingly recognized as a risk factor for type 2 MI and an important prognostic marker of future cardiovascular risk. The DEMAND-MI (Determining the Mechanism of Myocardial Injury and Role of Coronary Disease in Type 2 Myocardial Infarction) prospective cohort study applied systematic cardiac and coronary imaging in patients with a provisional diagnosis of type 2 MI and identified coronary artery disease in two-thirds of patients, which was previously unrecognized in the majority.^3^ This finding was independently validated in the DEFINE-MI (Determining the Prevalence and Characteristics of Coronary Artery Disease Among Patients with Type 2 Myocardial Infarction using CT-FFR) cohort study, where an even higher prevalence of coronary disease was observed,^4^^,^^5^ suggesting this substrate plays a key role in susceptibility for type 2 MI events. In this study, we found that almost half of patients with type 2 MI in TARGET-Type 2 reported antecedent anginal symptoms. This finding challenges the notion that ischemia is purely related to illness severity and suggests identification and treatment of angina may represent an important patient-focused target for treatment.

### Study strengths and limitations

One major strength of this study was its prospective design with inclusion of patient-reported outcome measures to capture patient experience. However, we recognize that this is a secondary analysis of a small pilot randomized controlled trial. Additionally, we were unable to follow-up for symptoms after their type 2 MI event. Therefore, our observations require validation in a larger more diverse population to ensure they are generalizable. We used the shortened rose angina questionnaire, but recognize novel, digitally supported approaches, such as use of the ORBITA symptom app, would minimize recall bias and permit more accurate recording. Longitudinal studies are required to address the progression of symptoms following a type 2 MI event and to evaluate whether traditional therapies for stable angina are effective in this multimorbid population. Addressing this gap in our evidence base will be crucial in defining patient-centered goals of treatment.

## Conclusions

In this pilot randomized controlled trial of patients with type 2 MI, nearly half reported anginal symptoms in the 3 months preceding their acute presentation. These findings suggest that myocardial ischemia in type 2 MI is not solely attributable to acute physiological stress but may reflect untreated symptomatic coronary artery disease in a substantial proportion of patients. Systematic assessment of symptoms before and after type 2 MI may identify patient-centered targets for investigation and treatment. Longitudinal studies incorporating symptom burden alongside imaging and clinical outcomes are now required to determine whether targeted therapies can improve quality of life and cardiovascular outcomes in this high-risk population.

## Funding support and author disclosures

This work was supported by Chief Scientist Office (Scotland) TCS 22/31. The authors have reported that they have no relationships relevant to the contents of this paper to disclose.
